# Nest size is predicted by female identity and the local environment in the blue tit (*Cyanistes caeruleus*), but is not related to the nest size of the genetic or foster mother

**DOI:** 10.1098/rsos.172036

**Published:** 2018-04-18

**Authors:** Louis G. O'Neill, Timothy H. Parker, Simon C. Griffith

**Affiliations:** 1 Department of Biological Sciences, Macquarie University, Sydney, New South Wales 2109, Australia; 2 Edward Grey Institute, Department of Zoology, University of Oxford, Oxford OX1 3PS, UK; 3 Department of Biology, Whitman College, Walla Walla, WA 99362, USA

**Keywords:** cross fostering, nesting behaviour, cavity nest, heritability, micro-environment, parental effects

## Abstract

The potential for animals to respond to changing climates has sparked interest in intraspecific variation in avian nest structure since this may influence nest microclimate and protect eggs and offspring from inclement weather. However, there have been relatively few large-scale attempts to examine variation in nests or the determinates of individual variation in nest structure within populations. Using a set of mostly pre-registered analyses, we studied potential predictors of variation in the size of a large sample (803) of blue tit (*Cyanistes caeruleus*) nests across three breeding seasons at Wytham Woods, UK. While our pre-registered analyses found that individual females built very similar nests across years, there was no evidence in follow-up (*post hoc*) analyses that their nest size correlated to that of their genetic mother or, in a cross-fostering experiment, to the nest where they were reared. In further pre-registered analyses, spatial environmental variability explained nest size variability at relatively broad spatial scales, and especially strongly at the scale of individual nest boxes. Our study indicates that nest structure is a characteristic of individuals, but is not strongly heritable, indicating that it will not respond rapidly to selection. Explaining the within-individual and within-location repeatability we observed requires further study.

## 1. Introduction

Most bird species construct nests in which to lay eggs and raise young [1], and the qualities of the nest often influence reproductive success [2]. Nest construction and structure varies significantly between species, with major structural differences, such as the presence or absence of roofs having emerged in ancestral groups as long as 40 million years ago [3]. However, even within a species there remains extensive variation in the structural qualities of nests that has received relatively little attention, although it has been the focus of recent studies investigating the cognitive processes underlying the nest building process [4]. Nest building is a complicated behaviour with high levels of context-dependency, but how nest-building decisions are made is poorly understood [5]. For instance nest dimensions of southern masked weavers *Ploceus velatus*, showed low within-male repeatability [6], but more experienced males built neater, tighter nests [7]. While nest structures may differ across individuals within a population in line with factors such as condition or other intrinsic attributes of the nest-building parent, there are limited large-scale data looking at the extent to which nest variation is determined by additive genetic variation, or environmental parameters that might relate to nest structure directly or indirectly (but see [8,9]).

Nest building may attract the attention of predators [10], and also requires increased activity and thus energy consumption, and therefore individual variation in condition or resources might influence nest size. For instance, experimental provisioning with supplementary food during nest building led to construction of larger nests by blue tits *Cyanistes caeruleus* in one study [11] and shallower nests, built more quickly in another [12]. Manipulations that increase the effort required to build a nest can increase stress [13] and negatively impact reproductive behaviours such as incubation, provisioning and clutch size [14–16]. The costliness of nest construction has led some to hypothesize that nests are honest signals of condition, and even a sexually selected trait [17,18].

To investigate potential sources of variation in the nest size (recorded as nest depth, and previously referred to as the ‘structural layer’ [19]) in a natural population, we studied the blue tit in Wytham Woods, UK. They are locally abundant and readily nest in artificial nest boxes [20], allowing a large sample in controlled nesting conditions. Nests are built solely by females in this species [21] and consist of a thick layer of moss and other plant material with a tightly woven cup of hair, feathers and fine grasses sunk into the top of the moss layer [11]. There is a high degree of adult and offspring philopatry in this population enabling us to investigate nest structure within a female across multiple contexts and also to compare the nest of mothers with the nests subsequently produced by their daughters in later years. Furthermore, in this well-studied site, there is significant, and well-characterized environmental variation across different compartments of the woodland which affects fledgling condition [22], clutch size and clutch mass [23], and adult fecundity [24]. We hypothesized that variation in nest size would be due to intrinsic differences among females, variation in characteristics of the environment, or both. We tested these hypotheses by exploring consistency within a female in nest size across years, mother–daughter correlations in nest size, within-nest box consistency in nest size across years, and differences in nest size among compartments of the study site.

## 2. Material and methods

### 2.1. Field methods

We collected nest size data (recorded as nest depth using the fraction of nest box filled) which accounts for nest height or the ‘structural layer’ [19] during the breeding seasons of 2001–2003 in Wytham Woods, Oxfordshire, UK (1°20′ W, 51°47′ N). The date of first egg laying was determined for all but one nest through regular nest box checks during the nesting season. Nest sizes were recorded within 3 days of clutch completion, with all data collected by one observer (SCG). In total, 873 nests were measured; however, this included nests in two different types of nest boxes (Schwegler 2M with 32 and 26 mm entrance holes and slightly different internal dimensions) [25]. The nest boxes with a 26 mm entrance hole were only installed in the final year of our study (2003). As these nest boxes had slightly different internal dimensions, and only 70 nests were constructed in these boxes in our total dataset, we removed these from our analyses, leaving a total sample size of 803 nests in standardized boxes. As this issue was first brought to our attention by a reviewer, we had previously conducted the same analyses with these nest boxes included. That previous analysis can now be seen in appendix 1, and the findings are consistent across both analyses.

The female owner of the nest was determined later, when both parents were caught while feeding the nestlings in that nest. Parents were trapped in the nest box and were fitted with a uniquely numbered ring supplied by the British Trust for Ornithology (BTO), under UK Home Office licence. Nestlings were ringed when they were 14 days old and the rings allowed us to track individuals across years and tie them to additional nests later in life.

In addition to measuring the nests of all blue tits breeding in Wytham in these years, a cross-fostering experiment was carried out on a large subset of all of the nestlings, allowing the partial segregation of rearing environment effects from assessment of heritability of nest size. In the cross-fostering experiment, at least half of the nestlings in each nest were swapped with those from another nest at about 2–3 days old. Across the 3 years, nestlings were cross-fostered in a total of 705 out of 907 broods in Wytham, although we did not record nest size for all of these broods. The cross-fostering meant that many individuals were raised by foster parents, allowing for the comparison of many females' nests with the nests of both their genetic mother and their foster mother.

We investigated potential effects of the environment at two different scales. Because the location of nest boxes was identical from year to year, we could assess the effects of the immediate environment on nest size by assessing consistency of nest size within individual nest boxes. We considered environmental effects at a larger scale by examining variation in nests across nine different compartments (mean 45 ha, minimum 18 ha) of Wytham Woods. These different compartments have been previously well defined and they are biologically relevant, based on the age and species composition of trees, and known to affect clutch size and mass and also fledgling condition [22,23]. All data are available on Dryad Digital Repository http://dx.doi.org/10.5061/dryad.sc30g [26].

### 2.2. Statistical analysis

**Figure 1.**
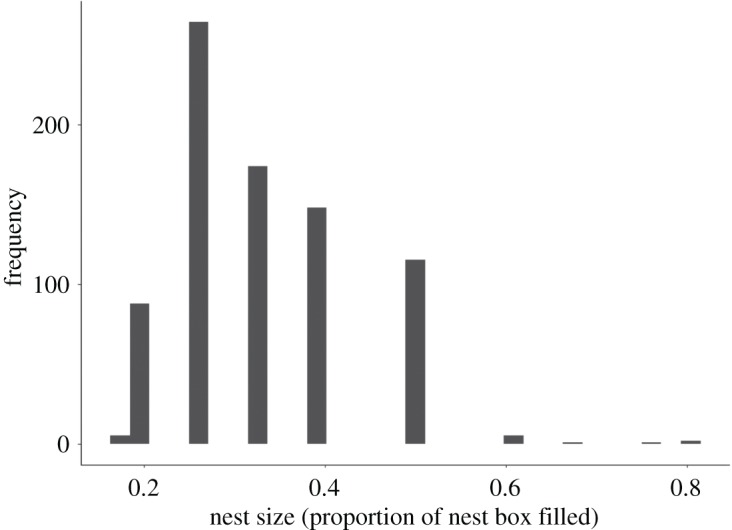
The distribution of nest sizes, which were estimated as the proportion of the nest box filled with nesting material, observed in this study of blue tits at Wytham Woods, UK (*n* = 803).

We carried out all analysis in R [27]. We pre-registered an analysis plan (doi:10.17605/OSF.IO/MJEHR) prior to examining the data to avoid practices that inflate type I error [28]. In accordance with our pre-registration, we initially converted our nest size proportions into six ordinal categories (1 = ≤0.2 (*n* = 101), 2 = 0.25 (*n* = 304), 3 = 1/3 (*n* = 187), 4 = 0.4 (*n* = 154), 5 = 0.5 (*n* = 118), 6 = ≥0.6 (*n* = 9)) because of the non-normal distribution of these proportions (figure 1). We then conducted cumulative link mixed effects models (CLMMs) in the Ordinal package [29] (using Laplace approximations to generate *p*-values) to investigate the effects of different variables on nest size (treated as ordinal categories). Unfortunately, these analyses produced a Hessian singularity in some of our models. Thus we had to deviate from our analysis plan and instead opted to model nest size based on the raw proportions as a continuous variable. We did this with linear mixed effects models (LMMs) in the lme4 package [30] using Wald tests to create *p*-values for linear models and Satterthwaite's approximations in linear mixed effects models, in the lmerTest package [31]. Unfortunately our data do not fit within any of the available error distributions in a GLMM and could not be transformed to fit one either. Although linear models are relatively robust to deviations from normality [32], our tests of significance should be viewed as tentative, especially in cases of marginal significance.

As laid out in our pre-registration, the initial analyses were designed to investigate any relationship between nest size and both female identity and nest box identity. We compared nest size to female identity in two analyses. In the first, we used all females that bred more than once (*n* = 223 nests by 105 females, 86 of which bred twice and 17 bred three times), and in the second, we limited our data to females that bred more than once, each time in a different nest box (*n* = 149 nests by 79 females (70 females bred twice in different boxes and nine females that bred more than three times but only once in a different box compared to their other nests)). Similarly, when comparing nest size to nest box identity, we first used all nest boxes occupied more than once (*n* = 444 nests in 201 boxes, 159 were used twice and 42 were used in all three seasons), and then in a second analysis, limited the data to boxes that had been occupied more than once, each time by a different female (*n* = 107 nests in 57 boxes of which three nest boxes had a different female in all 3 years, 44 nest boxes had different females in 2 years and 10 had a different female in only 1 year (although they were used in multiple years)). In our final assessment of female identity and nest box identity relationships with nest size, we limited our analysis to only those females that bred more than once, each time in a nest box that had been, or would be used more than once, to provide estimates of female identity and nest box identity effects that account for the other (*n* = 148 nests). Next, we compared nest size to date category (early versus late) of first egg laid (*n* = 516 nests) as a fixed effect. We categorized dates into early, mid or late categories for each year to create approximately equal sample sizes in each category (257 early, 286 mid, 259 late and one nest excluded because clutch initiation date unknown). We then compared nest sizes between early and late nests, ignoring the middle third to avoid comparing nests that were very similar in laying date. A further analysis investigated if a change across years in relative lay date of the first egg by a female was correlated with a change in the size of her nest across those years to investigate the idea that nest size might be a function of the duration of time a female has to build before she starts laying. For this analysis we standardized lay date across years by setting the first egg date of each year as day one and then z-transformed laying date (by removing the mean from the lay date, then dividing by the standard deviation) [33]. We also compared nest size to compartment in the woodland and year (*n* = 803 nests), both as fixed effects. In all but the last of these models, we included year and compartment as random effects to account for any effect they may have on nest size. A further analysis (not pre-registered) was conducted at the suggestion of a reviewer in which nest box was nested within woodland compartment and included year as a fixed effect (*n* = 803 nests). The model enabled us to investigate the effect of compartment while accounting for the effect of nest box.

Results from the analyses described above indicated repeatability of nest size within females (see Results), and so we conducted follow-up (not pre-registered) analyses to investigate the possibilities that nest size might be heritable or might be influenced by the size of nest in which a female was reared. We first investigated the extent to which a female's nest size was predicted by the nest size of her foster mother (*n* = 40 foster mother–daughter pairs in a cross-fostering experiment, see [24]). After we failed to find an effect of foster mother (see Results), we compared female nest size with the nest size of their genetic mother (regardless of cross-fostering; *n* = 70 mother–daughter pairs).

## 3. Results

In total we measured 803 nests in the standardized nest box design from a total of 596 different females in 629 nest boxes; however, sample sizes for most of our analyses are subsets of these 803 (as detailed in table 1).

**Table 1.** Explanations for the sample sizes used in our analyses. Sample sizes are numbers of individual nests unless otherwise noted.

	sample size				
analysis	total	2001	2002	2003	sample size explanation
female identity	223	70	102	51	Only includes females that bred more than once (86 females bred in 2 years, 17 females bred all 3 years)
females in different nest boxes	149	46	69	34	Only includes multiple-breeding females for which each nest was in a different nest box (70 females bred twice in different boxes and nine females bred more than three times but only once in a different box compared to their other nests)
box identity	444	143	157	144	Only includes nest boxes that were used more than once (159 were used twice and 42 were used in all 3 years)
nest boxes with different females	107	31	38	38	Only includes nest boxes that were used more than once by more than one female (three nest boxes were used in all 3 years, two were used in 44 years and 10 nest boxes were used more than once but in only 1 year by a different female)
combined female and nest box identity	148	46	72	30	Only includes females that bred more than once, each time in a nest box that had been or would be used more than once
lay date	516	164	174	178	Only includes nests were the first egg was laid in the first or last third of each season
woodland compartment	803	250	273	280	The full dataset in standardized nest box design
year	803	250	273	280	The full dataset in standardized nest box design (250 of which were in 2001, 273 were in 2002 and 280 were in 2003)
rearing nest similarity	40	18	22	0	The number of females with a measured nest who were reared by a foster mother with a measured nest.
genetic mother similarity	70	29	41	0	The number of females with a measured nest for whom the putative genetic mother had a measured nest. This included both cross-fostered and natal-fostered chicks because we found no relationship between rearing nest size and adult nest size.
lay-change	223	70	102	51	Differences in laying date of nests by repeat breeding females

**Figure 2.**
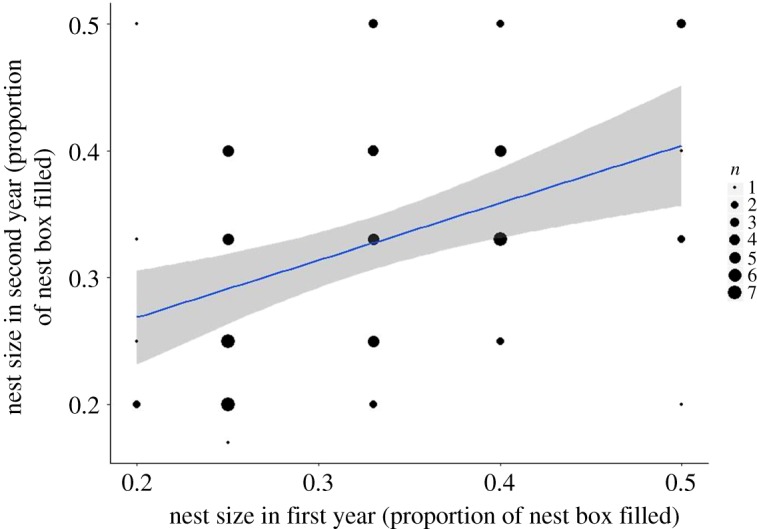
The relationship of the size of a second nest as a function of the size of a previous year's nest in blue tits (*Cyanistes caeruleus*) in Wytham Woods, Oxfordshire, UK. We collected data in April–May 2001–2003. These data are from females that bred more than once in the study period and used different nest boxes in each nesting attempt, *n* = 149 nests from 79 females. The line of best fit represents the variation explained by female identity as calculated from linear models with and without female identity included, *χ*^2^ = 7.55, *p* = 0.006, *R*^2^ = 0.615; the grey shaded area shows 95% confidence interval.

Female identity showed a highly significant relationship with nest size (LMM_223_, *χ*^2 ^= 21.48, *p* < 0.001), as shown in table 2. The model containing just female identity with year and wood compartment as random effects explained a significant amount of variation (*R*^2 ^= 0.62). When females breeding repeatedly in the same nest box were removed to avoid any confounding effect of nest box identity, the explanatory value of the model remained significant *R*^2^ = 0.62 and female identity also retained statistical significance (*χ*^2 ^= 7.55, *p* = 0.006, *n* = 149 attempts by 79 females) (table 3, figure 2).

**Table 2.** Results from a linear mixed effects model examining predictors of nest sizes of blue tits (*Cyanistes caeruleus*) in Wytham Woods, Oxfordshire, including year, compartment and female identity. This analysis was run on a subset of data where females bred more than once in the study period (*n* = 223 nests and 103 females). This model had an *R*^2^ of 0.621.

	estimate ± s.e.		*T*-value	
intercept	0.321 ± 0.017		19.08	
random effect		Chi d.f.	*χ*^2^	*p*
female ring number		1	21.48	4 × 10^−6^
compartment		1	9.65	0.0012
year		1	0.62	0.430

**Table 3.** Results from a linear mixed effects model examining predictors of nest sizes of blue tits (*Cyanistes caeruleus*) in Wytham Woods, Oxfordshire, including compartment, year and female identity. This analysis was run on a subset of data where females bred more than once in the study period but did not reuse the same nest box (*n* = 149 nests and 79 females). This model had an *R*^2^ of 0.615.

	estimate ± s.e.		*T*-value	
intercept	0.329 ± 0.019		17.14	
random effect		Chi d.f.	*χ*^2^	*p*
female ring number		1	7.55	0.006
compartment		1	6.18	0.013
year		1	0.01	0.905

**Figure 3.**
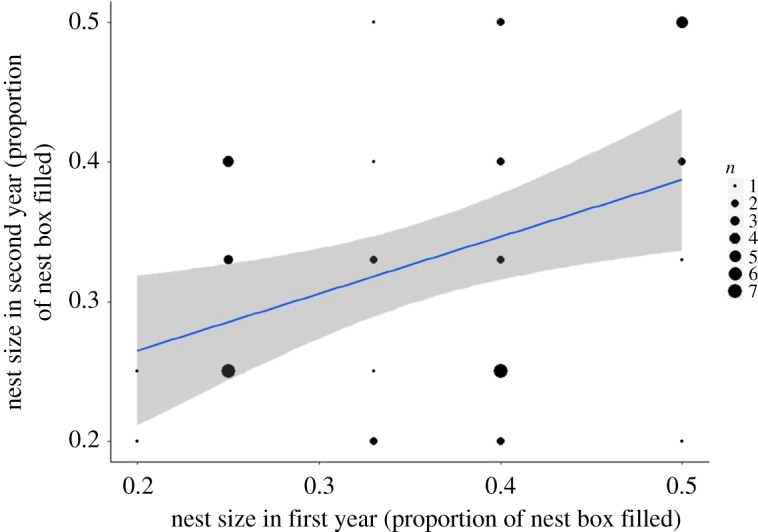
The relationship of nest size of a second nest compared to the nest from previous years in the same nest box by blue tits (*Cyanistes caeruleus*) in Wytham Woods, Oxfordshire, UK. Data were collected in April–May 2001–2003. These data are from nest boxes which were used more than once in the study period by different females, *n* = 107 nests in 57 nest boxes. The line of best fit represents the variation explained by a linear mixed effects model including box identity, compartment and year, *χ*^2^ = 23.07, *R*^2^ = 0.265; the grey shaded area shows 95% confidence interval.

Nest box identity, which is a surrogate for microhabitat, showed a moderate but significant relationship with nest size (*χ*^2 ^= 11.07, *p* < 0.001, *n* = 444 attempts in 201 different nest boxes) as shown in table 4, the model of just nest box identity with year and forest compartment as random effects generated an *R*^2^ of 0.39, when we analysed nest boxes that were used more than once. By removing nest boxes used more than once by the same female, but leaving nest boxes used by different females (leaving us to examine 107 breeding attempts in 57 nest boxes of which three nest boxes had a different female in all 3 years, 44 nest boxes had different females in 2 years and 10 had a different female in only 1 year (although they were used in multiple years)) this relationship was weaker, and nest box identity was no longer statistically significant (*R*^2 ^= 0.27, *χ*^2 ^= 0.779, *p* = 0.4, table 5, figure 3).

**Table 4.** Results from a linear mixed effects model examining factors affecting nest sizes of blue tits (*Cyanistes caeruleus*) in Wytham Woods, Oxfordshire. This analysis was run on a subset of data where nest boxes were used more than once in the study period (*n* = 444 nests in 201 nest boxes). This model had an *R*^2^ of 0.388.

	estimate ± s.e.		*T*-value	
intercept	0.331 ± 0.012		27.06	
random effect		Chi d.f.	*χ*^2^	*p*
box number		1	11.07	9 × 10^−4^
compartment		1	14.18	2 × 10^−4^
year		1	0.79	0.400

**Table 5.** Results from a linear mixed effects model examining factors affecting nest sizes of blue tits (*Cyanistes caeruleus*) in Wytham Woods, Oxfordshire. This analysis was run on a subset of data where nest boxes were used more than once in the study period but not by the same female (*n* = 107 nests in 57 nest boxes). This model had an *R*^2^ of 0.265.

	estimate ± s.e.		*T*-value	
intercept	0.344 ± 0.015		23.07	
random effect		Chi d.f.	*χ*^2^	*p*
box number		1	0.779	0.4
compartment		1	0.819	0.4
year		1	0.000	1.0

When we included female identity and nest box identity in the same model to investigate the extent to which the variation they explained in nest size was independent, both continued to explain significant portions of the variance, the model *R*^2^ = 0.88, (female identity: *χ*^2^ = 10.37, *p* = 0.001; nest box identity: *χ*^2^ = 10.73, *p* = 0.001, table 6).

**Table 6.** Results from a linear mixed effects model examining factors affecting nest sizes of blue tits (*Cyanistes caeruleus*) in Wytham Woods, Oxfordshire. This analysis was run on a subset of data where nest boxes were used more than once in the study period by females that bred more than once (*n* = 148 nests from 87 females in 99 nest boxes). This model had an *R*^2^ of 0.875.

	estimate ± s.e.		*T*-value	
intercept	0.327 ± 0.018		18.02	
random effect		Chi d.f.	*χ*^2^	*p*
box number		1	10.73	0.001
ring number		1	10.37	0.001
compartment		1	4.70	0.030
year		1	0.72	0.395

**Figure 4.**
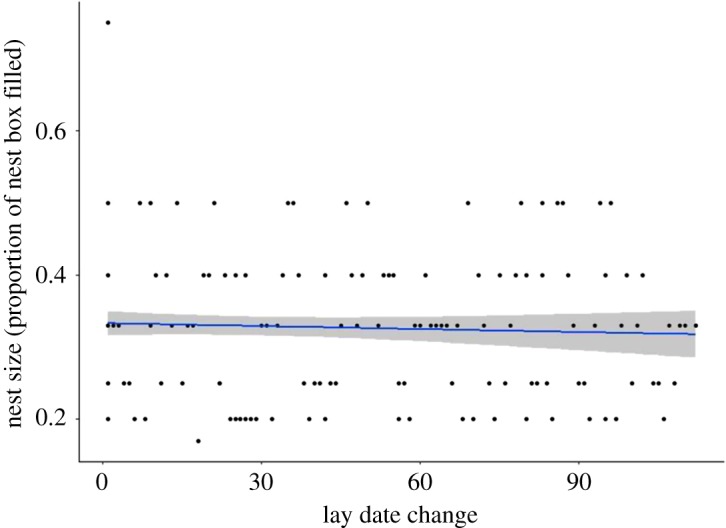
The relationship of nest size and difference in date of first egg laid by repeat-breeding blue tits (*Cyanistes caeruleus*) in Wytham Woods, Oxfordshire, UK. Data were collected in April–May 2001–2003. These data are a subset of data from females that bred more than once, to investigate any effect their difference in lay date has on their nest size (*n* = 223). The *R*^2^ shows a model of lay-date change was 0.176, although this was not statistically significant (LMM_223_ *T *= −2.012, *p* = 0.313); the grey shaded area shows 95% confidence interval.

In another analysis conducted to explore drivers of within-female consistency, we found a weak relationship with first egg lay date such that the last third of initiated nests were on average just 2.1% of a nest box smaller than the first third of initiated nests (34.2% ± 10.0%, 32.1% ± 10.0% (LMM_516_, *p* = 0.03, *n* = 516, table 7)). However, when we compared the multiple nests of females that bred more than once and compared the difference in date of clutch initiation between their first and second attempt with the size of the resulting nest, we found no evidence that females who moved their laying date later or earlier made their nests smaller or larger (LMM_223_ *p* = 0.313, *R*^2^ = 0.18, *n* = 223, table 8 and figure 4).

**Table 7.** Results from a linear mixed effects model examining factors affecting nest sizes of blue tits (*Cyanistes caeruleus*) in Wytham Woods, Oxfordshire. This analysis was run on a subset of data of breeders that initiated laying in the first and last third of each season (*n* = 516); degrees of freedom were calculated from Satterthwaite approximations. This model had an *R*^2^ of 0.076.

parameter	estimate ± s.e.	d.f.	*T*-value	*p*
intercept	0.342 ± 0.010	11.20	33.102	1.44 × 10^−12^
lay date category	−0.021 ± 0.010	401.80	−2.17	0.030
random effect		Chi d.f.	*χ*^2^	*p*
compartment		1	9.21 × 10^0^	0.002
year		1	1.14 × 10^−13^	1.000

**Table 8.** Results from a linear mixed effects model examining factors affecting nest sizes of blue tits (*Cyanistes caeruleus*) in Wytham Woods, Oxfordshire. This analysis was run on a subset of data of females that bred more than once to investigate any effect their difference in lay date has on their nest size (*n* = 223); degrees of freedom were calculated from Satterthwaite approximations. This model had an *R*^2^ of 0.176.

parameter	estimate ± s.e.	d.f.	*T*-value	*p*
intercept	3.254 × 10^−1^ ± 1.762 × 10^−2^	7.480 × 10^0^	18.467	1.63 × 10^−7^
change in lay date	−1.731 × 10^−4^ ± 1.712 × 10^−4^	2.121 × 10^2^	−1.012	0.313
random effect		Chi d.f.	*χ*^2^	*p*
compartment		1	1.9 × 10^1^	1 × 10^−5^
year		1	5.68 × 10^−14^	1.000

**Figure 5.**
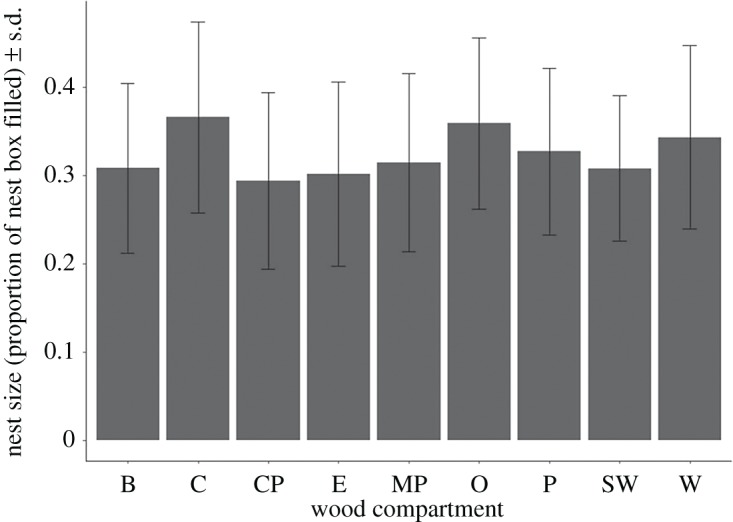
Size of blue tit (*Cyanistes caeruleus*) nests in the different compartments of Wytham Woods, Oxfordshire, UK. Nest depth averages recorded as proportion of nest box filled ± s.d. for each compartment: B 0.308 ± 0.964, C 0.366 ± 0.108, CP 0.279 ± 0.079, E 0.302 ± 0.104, MP 0.315 ± 0.101, O 0.348 ± 0.098, P 0.315 ± 0.088, SW 0.308 ± 0.083, W 0.343 ± 0.104. Data were collected in April–May 2001–2003. These data are from all recorded breeding attempts (*n* = 803) in standardized boxes during the 2001–2003 breeding seasons.

The model investigating the effect of wood compartment and year had a conditional *R*^2^ of 0.057; compartment within the study site had a weak but statistically significant relationship with nest size (*F*_10,792 _= 5.887, *p* < 0.001) see table 9 and figure 5. When nest boxes were nested within compartments, the effect of compartment within the model remained significant; however, the model explained more of the variance (LMM_2,801_ *p* < 0.001, *R*^2^ = 0.40). This result indicates that the box ID accounted for most of the variance that this model explains, as this variance wasn't accounted for when box ID was not included in the model (table 10). Nest size also varied among years; however, this effect was weaker than that for compartment and was not quite statistically significant (*F*_2,801_ = 2.555, *p* = 0.078, *R*^2^ = 0.069, table 11).

**Table 9.** Results from a linear mixed effects model examining differences in the nine ‘compartments’ or geographical areas at Wytham Woods, Oxfordshire, UK and differences among the 3 years in nest sizes of blue tits (*Cyanistes caeruleus*) (*n* = 803 nests); degrees of freedom were calculated from Satterthwaite approximations; *p*-values were calculated from Wald tests. This model had an *R*^2^ of 0.057.

parameter	estimate ± s.e.	*T*-value	*p*
intercept	0.318 ± 0.010	30.985	<2 × 10^−16^
	compartment:^a^		
C	0.056 ± 0.013	4.447	9.97 × 10^−6^
CP	−0.015 ± 0.020	−0.746	0.456
E	−0.006 ± 0.012	−0.508	0.612
MP	0.006 ± 0.014	0.414	0.679
O	0.0507 ± 0.012	4.143	3.79 × 10^−5^
P	0.019 ± 0.026	0.752	0.452
SW	−0.001 ± 0.014	−0.62	0.950
W	0.034 ± 0.013	2.654	0.008
year:^b^			
2002	−0.019 ± 0.009	−2.222	0.027
2003	−0.008 ± 0.008	−0.916	0.360

^a^Reference category is B.

^b^Reference category is 2001.

**Table 10.** Results from a linear mixed effects model with nest boxes nested in woodland compartment to investigate the differences in the nine ‘compartments’ or geographical areas at Wytham Woods, Oxfordshire UK and differences among the 3 years in nest sizes of blue tits (*Cyanistes caeruleus*) (*n* = 803 nests); degrees of freedom were calculated from Satterthwaite approximations; *p*-values were calculated from Wald tests. This model had an *R*^2^ of 0.404.

parameter	estimate ± s.e.	d.f.	*T*-value	*p*
intercept	7.015 ± 8.449	644.40	0.830	0.407
year	−0.003 ± 0.004	644.30	−0.791	0.429
nested random effect		Chi d.f.	*χ*^2^	*p*
compartment : nest box ID		1	17.7	3 × 10^−5^

**Table 11.** Results from a linear mixed effects model examining the effect of inter-year variation, with forest compartment included as a random effect, on nest sizes of blue tits (*Cyanistes caeruleus*) in Wytham Woods, Oxfordshire (*n* = 803); *p*-values were calculated from Wald tests. This model had an *R*^2^ of 0.079.

parameter	estimate ± s.e.	*T*-value	*p*
intercept	0.336 ± 0.010	32.394	3.11 × 10^−15^
year:^a^			
2002	−0.020 ± 0.009	−2.257	0.024
2003	−0.008 ± 0.009	−0.895	0.371
random effect	Chi d.f.	*χ*^2^	*p*
compartment	29.9	1	4 × 10^−8^

^a^Reference category is 2001.

**Figure 6.**
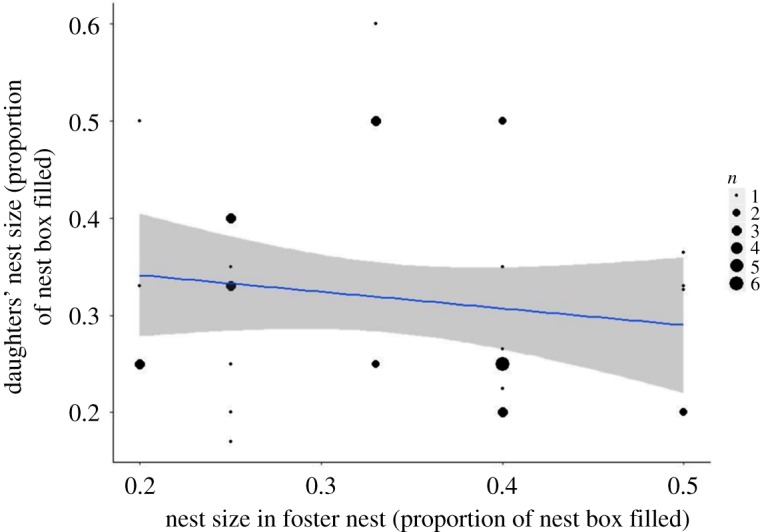
The relationship of foster mothers' and daughters’ nest size of blue tits (*Cyanistes caeruleus*) in Wytham Woods, Oxfordshire, UK. Data were collected in April–May 2001–2003. These data are from cross-fostered nestlings–foster mother pairs that both bred in the study system, *n* = 40. The line of best fit represents a linear model with *R*^2^ = 0.022, the grey shaded area shows 95% confidence interval.

**Figure 7.**
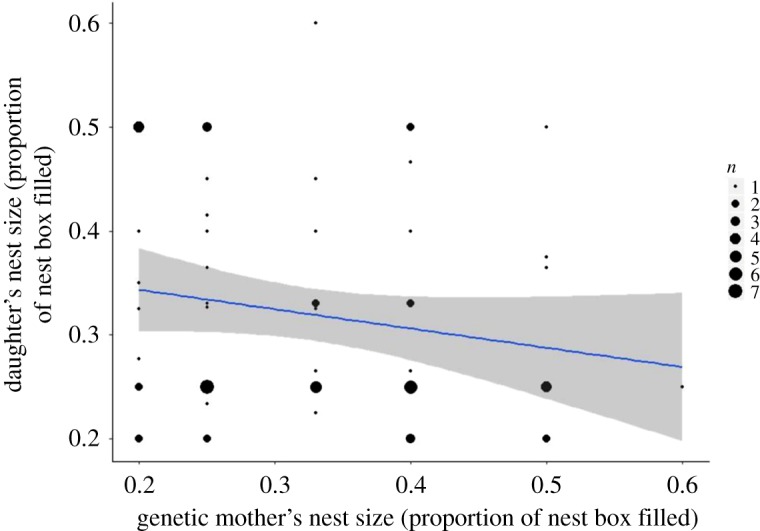
The relationship of genetic mothers' and daughters’ nest size of blue tits (*Cyanistes caeruleus*) in Wytham Woods, Oxfordshire, UK. Data were collected in April–May 2001–2003. These data are from genetic mother–daughter pairs that both bred in the study system, *n* = 70. The line of best fit represents a linear model with *R*^2^ = 0.033; the grey shaded area shows 95% confidence interval.

In our exploratory analyses conducted in response to consistency in nest size within matrilines, a daughter's nest size was not correlated with the nest size of her foster mother when cross-fostered (LMM_1,38_ *F* = 0.859, *p* = 0.36, *R*^2^ = 0.022, *n* = 40, table 12 and figure 6). We also found no evidence that nest size was heritable as the relationship of daughter's nest size to the genetic mother's nest size had an *R*^2^ of just 0.033 (*n* = 70) (LMM_1,68_ *F* = 2.289, *p* = 0.14, table 13 and figure 7).

**Table 12.** Results from a LMM examining the similarity of nest sizes of recruits to their foster mothers in blue tits (*Cyanistes caeruleus*) in Wytham Woods, Oxfordshire. This analysis was run on all cross-fostered daughters that proceeded to breed within the study site (*n* = 40). This model had an *R*^2^ of 0.022.

parameter	estimate ± s.e.	*T*-value	*p*
intercept	0.380 ± 0.047	8.144	7.38 × 10^−10^
recruit depth	−0.129 ± 0.139	−0.927	0.360

**Table 13.** Results from a LMM examining the similarity of nest sizes of recruits to their genetic mothers in blue tits (*Cyanistes caeruleus*) in Wytham Woods, Oxfordshire. This analysis was run on all cross-fostered daughters that proceeded to breed within the study site (*n* = 70). This model had an *R*^2^ of 0.0326.

parameter	estimate ± s.e.	*T*-value	*p*
intercept	0.384 ± 0.039	9.829	1.1 × 10^−14^
recruit depth	−0.176 ± 0116	−1.513	0.135

## 4. Discussion

Nest size in our population of blue tits varied as a function of both individual female identity and the local environment. However, while nest size is a somewhat consistent characteristic of a female's phenotype, we are not in a position to conclude what determined the size of a female's nest. Our findings are very similar to the recent study by Järvinen *et al.* [8], who also found, using an animal model approach, that daughters did not build similar nests to their genetic mothers. Our results from the cross-fostering design that we have used complement this recent non-experimental study by Järvinen *et al.* [8], experimentally separating the potential effects of genes and environmental sources of variation. We also found that an individual's nest did not resemble that of their foster mother, and hence the nests they built did not resemble that in which they were reared. We found clear evidence that the local environment (at two scales) influenced the size of nests. Box identity had a significant effect on nest size, even when we considered multiple nests made by different users of a particular box, showing that attributes of the local microclimate or microhabitat may play a role in the determination of nest characteristics. On a larger scale, the area within the woodland had a weaker, but still significant effect, consistent with previous research that has indicated the effect of the broader environment on other elements of life-history (e.g. [22,34]). We found very little support for the idea that nest size varied with the seasonal timing of egg laying, with no clear difference between early and late nests. We had thought that perhaps if females delayed the laying of their clutch, they may add extra material into the nest in the additional time, but this appears to not be the case.

We failed to find a convincing relationship between seasonal timing and nest characteristics. This result is consistent with the recent laboratory study by Lambrechts & Caro [9] in which photoperiod and temperature were experimentally manipulated, and in which blue and great tits did not adjust the size of their nest in response to their treatment. Yet other studies have found seasonal relationships in some other blue tit populations [35,36]. However, many of these studies are based on low sampling. For example, when Britt & Deeming [37] looked at the effect of ambient temperature on nest structure their conclusions were based on only 21 nests split across 2 years (nine nests in one year and 12 in the other). When Mainwaring *et al.* [35] investigated latitude and temperature effects they examined seven populations, but only 10 nests were measured in each population. The larger-scale studies such as our own, and the more recent studies by Lambrechts *et al.* [34], who analysed 3228 nests from 15 locations, and Järvinen *et al.* [8] who examined 1010 nests across a single population from 10 years, provide more robust estimates of the variation within populations and at the individual level.

Another explanation for the differences in results that is becoming apparent in the growing literature on nests (particularly in the Paridae), may lie with the choice of nest variables. Other studies have investigated cup characteristics (e.g. [36]), nest weight [21] or variation in constituent materials [37] which may impact a nest's insulating capacity more than our measure of nest size does. In fact, failure to find seasonal progression in nest size further supports the hypothesis that nest height has a purpose other than insulation. Similarly, McGowan *et al*. [38] found that the size of long-tailed tit *Aegithalos caudatus* nests did not change with the progressing season, although the lining decreased as the season progressed and temperatures warmed.

We were unable to explain the consistency of nest size within females. It was neither detectably heritable nor apparently influenced by the nest in which a female was reared. Although this shows that learning during rearing does not appear to influence nest size, learning after leaving the natal nest may. For instance in the zebra finch *Taeniopygia guttata*, individual learning clearly influences nest building [39]. It could also be that non-heritable aspects of quality influence nest construction. Another explanation could be that nest building is not under sufficient continuous selection pressure [8], or in fact that maintaining plasticity is selected for. This would allow appropriately sized nests to be built in a range of nesting cavities, as shown by the varying nest sizes built by great tits [40] thereby increasing the number of nesting cavities potentially available [41]. A further hypothesis is female condition, as studied by Tomás *et al*. [42] when they found repeatability in blue tit nest depth within females across two seasons, and they attributed this to consistent variation in female parasite-load. Experimental work in blue tits also found evidence that nest size relates to female condition, with females receiving supplementary food (thus presumably in better condition) building heavier nests [11], whereas Smith *et al.* [12] found that supplementary food caused blue tits to spend less time building their nests, resulting in shallower nests; however, this result was not matched in the great tits in their study. Lambrechts & Caro [9] also found that food availability did not affect nest size in blue tits under laboratory conditions, highlighting the context dependency of the importance of food availability. In our study, we did not manipulate condition prior to nest building, nor do we have any potential measure of condition besides the controversial mass-controlled-for-tarsus-length. Thus we were not in a position to conduct a robust test of the hypothesis that nest size is condition dependent and so we opted not to pursue such a test with our existing data. However, we may expect that both female condition (or quality) is fairly repeatable from one year to the next [42,43], and that females also tend to nest relatively close to the same area from one year to the next (S. Griffith 2003, personal observation) and therefore our results are broadly consistent with this idea.

Given the low heritability that we found for nest size, it would appear that this trait will not readily respond to selection, this could explain why nest size remains so variable within our population. However, empirical evidence from a variety of related species suggests that nest size is only sometimes under strong selection [8]. Across the Paridae, nest size predicts reproductive success in some species and some studies (e.g. [44–46]), but not in others [15,47]. We need a better understanding of the functional relevance of our metric of nest size (nest height, as referred to as ‘structural layer’ or ‘outer shell’ [5,19]) before we can better understand when we might or might not expect it to impact reproduction, and indeed how we might expect it to respond to a changing climate (or not), one avenue of study that may prove fruitful would be to investigate the significance of nest height in humidity control as has been shown for nest walls [48].

The relationships between box identity and nest size, and between woodland compartment and nest size, suggest that environmental variability at small to moderate spatial scales influences blue tit nest building, with small-scale environmental conditions having a greater effect. However, our data provide no information regarding which particular components of the environment may be influencing the measured aspect of nest structure. We did find that nest box identity (fine-scale location) explained more variability than woodland compartment (broad-scale location). This may be because even though compartments differ from each other, they share features with each other and most are moderately heterogeneous, whereas conditions at a given nest box should be fairly consistent from year to year. The next steps are to identify the features of the environment that predict nest size and especially nest depth variation so that we can formulate hypotheses about how and why individual birds respond to these features when building nests. We can start the exploration by focusing on local-scale environmental features already known to correlate with blue tit site occupancy and clutch sizes [49].

In summary, our data suggest that both female identity and nest box identity may determine significant proportions of the variation in nest size within a population. However, the many experimentally cross-fostered offspring in our study permitted us to robustly reject the existence of either heritability of nest size or an effect of nestling learning on nest size. Thus the mechanism driving individual consistency in nest morphology remains to be discovered. Likewise, environmental factors play an integral role in controlling nest size on at least two geographical scales, but the components of environmental variation driving the construction of nests of different sizes remains unknown in our system.

## Permission to carry out fieldwork

This work was conducted under the appropriate UK Home Office licence.

## Data accessibility

All data and analysis code are available at Dryad Digital Repository: http://dx.doi.org/10.5061/dryad.sc30g [26].

## Appendix

Tables using both types of nestbox.

**Table 14.** Explanations for the sample sizes used in our analyses. Sample sizes are numbers of individual nests unless otherwise noted.

		sample size			
analysis	total	2001	2002	2003	sample size explanation
female identity	230	70	102	58	only includes females that bred more than once (85 females bred in two years, 20 females bred all three years)
females in different nest boxes	173	48	81	44	only includes multiple-breeding females for which each nest was in a different nestbox (79 females bred twice in different boxes and 5 females that bred three times but only once in a different box compared to their other nests)
box identity	444	143	157	144	only includes nest boxes that were used more than once (159 were used twice and 42 were used in all three years)
nest boxes with different females	247	77	88	82	only includes nest boxes that were used more than once by more than one female (eight nest boxes were used in all three years, 106 were used in two years and 11 nest boxes were used more than once but in only one year by a different female)
combined female and nest box identity	148	46	72	30	only includes females that bred more than once, each time in a nest box that had been or would be used more than once
lay date	568	164	174	230	only includes nests were the first egg was laid in the first or last third of each season
woodland compartment	873	250	273	350	the full dataset in all nest boxes
year	873	250	273	350	the full dataset in all nest boxes
rearing nest similarity	43	18	25	0	the number of females with a measured nest who were reared by a foster mother with a measured nest.
genetic mother similarity	74	29	45	0	the number of females with a measured nest for whom the putative genetic mother had a measured nest. This included both cross-fostered and natal-fostered chicks because we found no relationship between rearing nest size and adult nest size.
lay-change	230	70	102	58	differences in laying date of nests by repeat breeding females

**Table 15.** Results from a linear mixed effects model examining predictors of nest sizes of blue tits (*Cyanistes caeruleus*) in Wytham woods, Oxfordshire, including year, compartment and female identity. This analysis was run on a subset of data where females bred more than once in the study period (*n* = 230 nests and 105 females). This model had an *R*^2^ of 0.63.

	estimate ± s.e.		*T*-value	
intercept	0.321 ± 0.016		19.600	
random effect		Chi d.f.	*χ*^2^	*p*
female ring number		1	23.93	<0.001
compartment		1	8.88	0.003
year		1	0.61	0.436

**Table 16.** Results from a linear mixed effects model examining predictors of nest sizes of blue tits (*Cyanistes caeruleus*) in Wytham woods, Oxfordshire, including compartment, year and female identity. This analysis was run on a subset of data where females bred more than once in the study period but did not reuse the same nest box (*n* = 173 nests and 84 females), This model had an *R*^2^ of 0.573.

	estimate ± s.e.		*T*-value	
intercept	0.321 ± 0.019		17.100	
random effect		Chi d.f.	*χ*^2^	*p*-value
female ring number		1	9.84	0.002
compartment		1	7.54	0.006
year		1	0.34	0.560

**Table 17.** Results from a linear mixed effects model examining factors affecting nest sizes of blue tits (*Cyanistes caeruleus*) in Wytham woods, Oxfordshire. This analysis was run on a subset of data where nestboxes were used more than once in the study period (*n* = 444). This model had an *R*^2^ of 0.388.

	estimate ± s.e.		*T*-value	
intercept	0.331 ± 0.012		27.06	
random effect		Chi d.f.	*χ*^2^	*p*-value
box number		1	11.07	9 × 10^−4^
compartment		1	14.18	2× 10^−4^
year		1	0.79	0.400

**Table 18.** Results from a linear mixed effects model examining factors affecting nest sizes of blue tits (*Cyanistes caeruleus*) in Wytham woods, Oxfordshire. This analysis was run on a subset of data where nestboxes were used more than once in the study period but not by the same female (*n* = 258). This model had an *R*^2^ of 0.24.

	estimate ± s.e.		*T*-value	
intercept	0.335 ± 0.013		25.86	
random effect		Chi d.f.	*χ*^2^	*p*-value
box number		1	1.25	0.26
compartment		1	5.68	0.02
year		1	0.03	0.87

**Table 19.** Results from a linear mixed effects model examining factors affecting nest sizes of blue tits (*Cyanistes caeruleus*) in Wytham woods, Oxfordshire. This analysis was run on a subset of data where nestboxes were used more than once in the study period by females that bred more than once (*n* = 148). This model had an *R*^2^ of 0.861.

	estimate ± s.e.		*T*-value	
intercept	0.326 ± 0.017		19.25	
random effect		Chi d.f.	*χ*^2^	*p*-value
box number		1	10.01	0.002
ring number		1	9.77	0.002
compartment		1	4.47	0.034

**Table 20.** Results from a linear mixed effects model examining factors affecting nest sizes of blue tits (*Cyanistes caeruleus*) in Wytham woods, Oxfordshire. This analysis was run on a subset of data of breeders that initiated laying in the first and last third of each season (*n* = 568), d.f.s were calculated from Satterthwaite approximations. This model had an *R*^2^ of 0.070.

parameter	estimate ± s.e.	d.f.	*T*-value	*p*-value
intercept	0.338 ± 0.010	11.500	35.48	4.11 × 10^−13^
lay date category	−0.023 ± 0.009	369.900	−2.46	0.014
random effect		Chi d.f.	*χ*^2^	*p*-value
compartment		1	6.99	0.008
year		1	0.00	1.000

**Table 21.** Results from a linear mixed effects model examining factors affecting nest sizes of blue tits (*Cyanistes caeruleus*) in Wytham woods, Oxfordshire. This analysis was run on a subset of data of females that bred more than once to investigate any affect their difference in lay date has on their nest size (*n* = 230), d.f.s were calculated from Satterthwaite approximations. This model had an *R*^2^ of 0.013.

parameter	estimate ± s.e.	d.f.	*T*-value	*p*-value
intercept	0.312 ± 0.019	6.510	16.124	1.73 × 10^−6^
change in lay date	−0.126 ± 0.007	115.800	−1.806	0.073
random effect		Chi d.f.	*χ*^2^	*p*-value
compartment		1	1.36 × 10^1^	2 × 10^−4^
year		1	2.84 × 10^−14^	1

**Table 22.** Results from a linear mixed effects model examining factors affecting nest sizes of blue tits (*Cyanistes caeruleus*) in the different compartments of Wytham woods, Oxfordshire (*n* = 873) across three breeding seasons, d.f.s were calculated from Satterthwaite approximations, *p*-values were calculated from Wald tests. This model had an *R*^2^ of 0.071.

parameter	estimate ± s.e.	*T*-value	*p*-value
intercept	0.321 ± 0.010	31.871	2 × 10^−16^
compartment:^a^			
C	0.056 ± 0.012	4.515	7.21 × 10^−6^
CP	−0.029 ± 0.015	−1.881	0.060
E	−0.007 ± 0.012	−0.531	0.596
MP	0.006 ± 0.014	0.438	0.661
O	0.039 ± 0.011	3.417	6.62 × 10^−4^
P	0.006 ± 0.020	0.308	0.758
SW	−0.002 ± 0.014	−0.106	0.916
W	0.035 ± 0.013	2.698	0.007
year:^b^			
2002	−0.020 ± 0.009	−2.264	0.024
2003	−0.016 ± 0.008	−1.962	0.050

^a^Reference category: B.

^b^Reference category: 2001.

**Table 23.** Results from a linear mixed effects model examining the effect of inter-year variation with forest compartment included as a random effect on nest sizes of blue tits (*Cyanistes caeruleus*) in Wytham Woods, Oxfordshire (*n* = 873), *p*-values were calculated from Wald tests. This model had an *R*^2^ of 0.060.

parameter	estimate ± s.e.	*T*-value	*p*-value
intercept	0.334 ± 0.010	31.888	5.77 × 10^−15^
year:^a^			
2002	−0.020 ± 0.009	−2.296	0.022
2003	−0.017 ± 0.008	−2.017	0.044
random effect	Chi d.f.	*χ*^2^	*p*-value
compartment	33.7	1	6 × 10^−9^

^a^Reference category is: 2001.

**Table 24.** Results from a linear mixed effects model examining the similarity of nest sizes of recruits to their foster mothers in blue tits (*Cyanistes caeruleus*) in Wytham woods, Oxfordshire. This analysis was run on all cross-fostered daughters that proceeded to breed within the study site (*n* = 43). This model had an *R*^2^ of 0.006.

parameter	estimate ± s.e.	*T*-value	*p*-value
intercept	0.329 ± 0.045	7.296	6.32 × 10^−9^
recruit depth	0.006 ± 0.132	0.048	0.962

**Table 25.** Results from a linear mixed effects model examining the similarity of nest sizes of recruits to their genetic mothers in blue tits (*Cyanistes caeruleus*) in Wytham woods, Oxfordshire. This analysis was run on all cross-fostered daughters that proceeded to breed within the study site (*n* = 74). This model had an *R*^2^ of 5.71 × 10^−5^.

parameter	estimate ± s.e.	*T*-value	*p*-value
intercept	0.344 ± 0.036	9.456	2.97× 10^−14^
recruit depth	−0.067 ± 0.011	−0.629	0.531
